# A Review of the Mycotoxin Family of Fumonisins, Their Biosynthesis, Metabolism, Methods of Detection and Effects on Humans and Animals

**DOI:** 10.3390/ijms26010184

**Published:** 2024-12-28

**Authors:** Christian Kosisochukwu Anumudu, Chiemerie T. Ekwueme, Chijioke Christopher Uhegwu, Chisom Ejileugha, Jennifer Augustine, Chioke Amaefuna Okolo, Helen Onyeaka

**Affiliations:** 1 School of Chemical Engineering, University of Birmingham, Birmingham B15 2TT, UK; 2 Department of Microbiology, Federal University Otuoke, Otuoke 562103, Bayelsa State, Nigeria; teresauzodinma@gmail.com (C.T.E.); chijiokeuhegwu@gmail.com (C.C.U.); augustinejennifer3@gmail.com (J.A.); 3 School of Health and Life Sciences, Teeside University, Darlington TS1 3BX, UK; 4 Bioinformatics and Genomics Research Unit, Genomac Institute, Ogbomosho, Oyo State, Nigeria; 5 Lancaster Environment Center, Lancaster University, Lancaster LA1 4YQ, UK; c.ejileugha@lancaster.ac.uk; 6 Department of Science Laboratory Technology (Microbiology), Imo State Polytechnic, Omuma 474110, Imo State, Nigeria; 7 Department of Food Science and Technology, Nnamdi Azikiwe University, Awka 420110, Anambra State, Nigeria; ca.okolo@unizik.edu.ng; 8 FOCAS Research Institute, Technological University Dublin, D07 EWV4 Dublin, Ireland

**Keywords:** fumonisins, mycotoxins, detection methods, food safety, immunoassays, health

## Abstract

Fumonisins, a class of mycotoxins predominantly produced by *Fusarium* species, represent a major threat to food safety and public health due to their widespread occurrence in staple crops including peanuts, wine, rice, sorghum, and mainly in maize and maize-based food and feed products. Although fumonisins occur in different groups, the fumonisin B series, particularly fumonisin B1 (FB1) and fumonisin B2 (FB2), are the most prevalent and toxic in this group of mycotoxins and are of public health significance due to the many debilitating human and animal diseases and mycotoxicosis they cause and their classification as by the International Agency for Research on Cancer (IARC) as a class 2B carcinogen (probable human carcinogen). This has made them one of the most regulated mycotoxins, with stringent regulatory limits on their levels in food and feeds destined for human and animal consumption, especially maize and maize-based products. Numerous countries have regulations on levels of fumonisins in foods and feeds that are intended to protect human and animal health. However, there are still gaps in knowledge, especially with regards to the molecular mechanisms underlying fumonisin-induced toxicity and their full impact on human health. Detection of fumonisins has been advanced through various methods, with immunological approaches such as Enzyme-Linked Immuno-Sorbent Assay (ELISA) and lateral flow immunoassays being widely used for their simplicity and adaptability. However, these methods face challenges such as cross-reactivity and matrix interference, necessitating the need for continued development of more sensitive and specific detection techniques. Chromatographic methods, including HPLC-FLD, are also employed in fumonisin analysis but require meticulous sample preparation and derivitization due to the low UV absorbance of fumonisins. This review provides a comprehensive overview of the fumonisin family, focusing on their biosynthesis, occurrence, toxicological effects, and levels of contamination found in foods and the factors affecting their presence. It also critically evaluates the current methods for fumonisin detection and quantification, including chromatographic techniques and immunological approaches such as ELISA and lateral flow immunoassays, highlighting the challenges associated with fumonisin detection in complex food matrices and emphasizing the need for more sensitive, rapid, and cost-effective detection methods.

## 1. Introduction

Mycotoxins are a broad range of low-molecular-weight toxic secondary metabolites produced by the mycelial structure of filamentous fungi/moulds [1,2]. These metabolites are associated with health risks, especially in cases of chronic exposure with an increased risk of cancer, immune-toxic effects, and significant developmental health risks, including neural tube birth defects (NTDs) and stunting in children, with further evidence suggesting that some of these mycotoxins are teratogenic [3,4,5,6]. Currently, over 400 mycotoxins have been reported to be produced by several fungal species; however, a single fungus can produce different mycotoxin; for example, aflatoxin is produced by 16 different species, and *Aspergillus bertholletius* produces over 3 different mycotoxins [7]. Mycotoxins are classified based on several factors, including the producer organism, structural similarity, effect, agronomic, and public health importance [8]. Examples include zearalenone (ZEA), trichothecenes, ochratoxins (OTA), aflatoxins (AFT), and fumonisins (FUMs). These mycotoxins affect more than 25% of crops harvested globally every year, with far-reaching consequences to the agricultural and industrial sectors with regards to economic losses and public health [9]. Fumonisins are a family of structurally similar mycotoxins mainly produced by *Fusarium verticilliodes* and *Fusarium proliferatum*. However, *F. anthophilum*, *F. proliferatum*, *F. subglutinans*, *F. fujikuroi*, *F. nygamai*, *Aspergillus welwitschiae*, and *Aspergillus niger* have also been implicated in the production of fumonisins [10,11,12,13,14,15,16]. Fumonisins are major contaminants of maize and products made from maize and are causative agents of various plant, animal, and human diseases, such as neural tube defects and esophageal cancer in humans [17,18], porcine pulmonary edema in pigs [19], and equine leukoencephalomalacia in horses [20]. Fumonisins are toxic alone and in complex with other mycotoxins and are implicated in immune suppression, hepatotoxicity, nephrotoxicity, and chronic liver diseases [21,22,23]. Importantly, the International Agency for Research on Cancer has designated FB_1_ (the most abundant of the fumonisins) as a class 2B carcinogen (possibly carcinogenic to humans) [23].

This review explores the occurrence and factors that affect the presence of the fumonisins, the regulations guiding levels of fumonisins in foods, the effects of fumonisins in humans and animals, and finally the methodologies currently employed for their detection in foods and feeds.

## 2. Occurrence of Fumonisins

Fumonisins (FBs) are widely distributed over the world, occurring in different groups: A, B, C, and P and 28 structural analogues have been characterized since 1988, including FA_1_, FA_2,_ FA_3,_ PHFA_3a,_ PHFA_3b,_ HFA_3_, FAK_1_, FBK_1_, FB_1_, Iso-FB_1_, PHFB_1a_, PHFB_1b_, HFB_1_, FB_2_, FB_3_, FB_4_, FB_5_, FC_1_, N-acetyl-FC_1_, Iso-FC_1_, N-acetyl-iso-FC_1_, OH-FC_1_, N-acetyl-OH-FC_1_, FC_3_, FC_4_, FP_1_, FP_2_, and FP_3_ [24,25]. The Fumonisin B series is considered the most abundant family as it contains Fumonisin B_1_ (FB_1_) and Fumonisin B_2_ (FB_2_) reported to be the most common and toxic variants as it is most prevalent in maize and maize products, which is further reported as the most commonly affected host plant [26,27]. However, FB_1_ is reported to occur at over 70% of the sum of FB_1,_ FB_2,_ Fumonisin B_3_ (FB_3_), and 25% of the total number of fumonisins with a comparatively higher cytotoxicity [28,29,30]. Aside from fumonisins occurrence in free-forms, they have also been reported to form compounds that may not be detected by conventional analytical methods and are sometimes referred to as ‘masked’ or ‘hidden’ forms [31].

The major fumonisin-producing genera are *Fusarium* and *Aspergillus*. It is worthy of note that they both colonize different substrates and produce different toxin profiles. *Fusarium*, especially *Fusarium verticilliode*, *Fusarium proliferatum*, and related *Fusarium* spp., produces FB1, FB2, and FB3, with FB1 being the most abundant and toxic among them [32,33], and they occur as saprophytes in the soil and colonize the rhizospheres, thereby affecting above-ground and below-ground plants [34,35,36]. Grain crops (rye, wheat, oats, rice, barley, millet, and maize) are the most common foods affected by fumonisin, with higher susceptibility associated with maize and maize products [14,27,37]. Additionally, fumonisins have also been reported in beer, wine, sugarcane, animal feed, tropical fruits, and milk (Table 1) [38,39,40,41,42,43]. In contrast, the only fumonisin producing *Aspergillus* is *A. niger*, which produces FB2, FB4, and FB6. *A. niger* has a broad ecological range and is commonly found colonizing diverse substrates, such as grapes, coffee beans, and nuts, including peanuts. The diversity of substrates reflects its ecological adaptability and the different environmental conditions under which it produces fumonisins [44].

Food contamination by fumonisin is affected by agroclimatic conditions, and the levels of contamination vary due to the hemi-biotrophic and necrotrophic behavior of the pathogens, in which the plant pathogenic fungi initially grow and extract nutrients from the plant and then switch to killing the plant from the secretion of enzymes and phytotoxins, including mycotoxins. The common occurrence of fumonisin and geographical distribution as a region have been reported to affect the distribution of fumonisin [45]. High levels have been detected in Africa: South-Africa, Morocco, Cameroon, Ghana, Nigeria, Zambia, and Kenya, amongst others [46], American countries: USA, Canada, Brazil, Argentina [47,48,49,50], Asia: India, China, Thailand, Philippines [51,52,53,54], Europe: Italy, Portugal [55,56] related to high maize consumption.

**Table 1:** Occurrence of fumonisin producers and their production in different regions.

Occurrence	Fumonisin	Bacterial Strain	Region	Reference
Maize (*Zea mays* L.)	B_1_, B_2_ and B_3_	*F. verticilloides*	Italy	[56]
	B_1_, and B_2_	*F. verticilloides*	Malaysia	[57]
	B_1_	*F. verticilloides*	Texas	[47]
	B_1_, B_2_, B_3_, B_4_ and A_1_	*Fusarium* spp.	South Africa	[58]
	B_1_, and B_2_	*Fusarium* spp.	Mexico	[59]
	B_1_, B_2_ and B_3_	*F. verticilloides*	Hungary	[60]
Oat (*Avena sativa*)	B_1_, and B_2_	*Fusarium* spp.	Spain	[61]
	B_1_	*Fusarium* spp.	Czech	[62]
	B_1_, and B_2_	*Fusarium* spp.	Spain	[61]
Figs	B_1_,	*Fusarium* spp.	Turkey	[63]
	B_1_, B_2_, and A	*Framigenum*, *F. solani*, and *F. proliferatum*	Italy	[64]
Raw cow milk	B_1_, and B_2_	*Fusarium* spp.	Portugal	[55]
	B_1_, and B_2_	*Fusarium* spp.	Brazil	[48]
Sugarcane	*FUM1*	*F. verticilloides* and *F. proliferatum*	Philippines	[52]
Grape-wine (Red wine)	B_2_	*A. niger*	Italy	[42]
Animal feed	B_1_	*Fusarium* spp.	Ghana	[38]
Rice	B_1,_ B_2_ and B_3_	*F. fujikuroi*	Malaysia	[65]
	B_1_, and B_2_	*F. verticilloides*, *F. andiyazi*, *F. fujikuroi* and *F. proliferatum*	Africa and Asia origin	[66]
Soil	B_1_, and B_2_	*F. proliferatum*, *F. verticilloides*	Malaysia	[57]
Paddy and Wheat	B_1_, B_2_ and B_3_	*F. sambucinum*, *F. fujikuroi* and other *Fusarium* spp.	China	[51]
Sweet pepper	B_1_	*F. lactis*, *F. proliferatum*, *F. verticilloides*	Canada	[50]
Sorghum	B_1_	*F.proliferatum*, *F.thapsinum*, *F. equuiseti*, *F. andiyazi* and *F. sacchari*	India	[54]
Wine	B_2_, B_4_	*Aspergillus niger* and *Aspergillus welwitschiae*	Australia	[16]

B_1_: Fumonisin B_1_, B_2_: Fumonisin B_2_, B_3_: Fumonisin B_3_, B_4_: Fumonisin B_4_, A_1_: Fumonisin A_1_, *FUM1*: Fumonisin-producing gene.

## 3. Chemistry and Biosynthesis of Fumonisins

**Figure 1:**
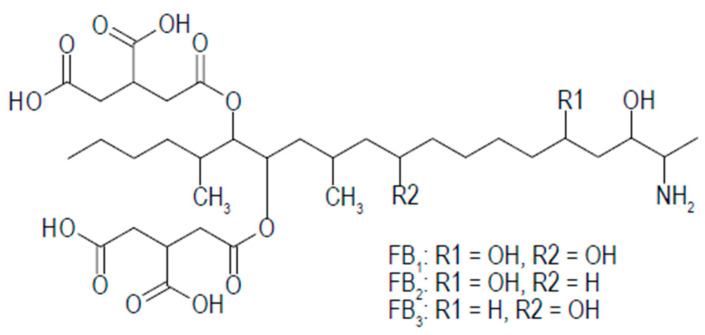
Chemical structures of the major fumonisins [68].

Fumonisins are polar compounds that are readily soluble in water and aqueous solutions of acetonitrile and methanol [67]. Fumonisins have a chemical structure composed of a 20-carbon amino polyhydroxy-alkyl chain di-esterified with propane-1,2,3-tricarboxylic acid (TCA), as shown in Figure 1 [68]. This chemical structure is similar to that of sphingosine (*So*) and sphinganine (*Sa*), and the toxicity of the fumonisins is suggested to be due to their structural analogy similar to the sphingoid bases and the inhibition of ceramide synthases (CerS), which leads to the accumulation of sphinganine [69,70]. Fumonisins act by inhibiting de novo sphingolipid biosynthesis and metabolism, resulting in an elevation of the serum ratio of sphinganine (*Sa*) and sphingosine (*So*) in the exposed animals [70,71]. Thus, the presence of these can serve as a biomarker of exposure to fumonisin and aid in their detection. In addition, other mechanisms of fumonisin toxicity and carcinogenicity have been proposed, including lipid peroxidation in laying hens [72], which is still in contention as the reports of [73] showed no substantial lipid peroxidation in piglets. Alteration of lipid biosynthesis and sphingolipids has also been reported [69].

The fumonisins are heat stable, being degraded only at high temperatures (>150 °C) utilized in baking, roasting, and extrusion. They can be stably present in the processing of foods and feeds and enter the food/feed chain, sometimes as covalently bound forms in heat-processed foods [67]. In a study carried out by Bullerman & Bianchini [74] to examine the stability of the fumonisins, a temperature of 175 °C for 60 min was necessary to achieve a 90% degradation of each fumonisin analog. The medium in which the fumonisin occurred did not affect the result of the experiment.

The biosynthesis of the fumonisins involves the “formation of linear demethylated polyketide and subsequent condensation of the polyketide with alanine. This condensation is followed by a carbonyl reduction, oxygenations, and esterification with two propane−1,2,3-tricarboxylic acids” [75]. In vitro studies have shown that production of fumonisins is crucially dependent on the presence of sugars (most importantly amylopectin), the pH of the medium, water activity, and nitrogen concentration in the medium [76].

The formation of fumonisin has been described as a complex process dependent on host-fungal pathogen interaction and agroclimatic factors. Fumonisin biosynthesis has been reported to be regulated by *FUM* gene clusters, and in recent times the ecological factors affecting the expressions of the genes are considered [77]. The *FUM* cluster, made up of 17 genes active in the biosynthesis of fumonisins, has been identified and characterized in *F. verticilliodes, F. oxysporum*, and *F. proliferatum*. *FUM1*, *FUM6*, *FUM8*, and *FUM21* play major roles in the synthesis of fumonisin, and FUM19 encodes an ATP-binding cassette protein that functions in transport mediation [78,79]. Additionally, FUM8 encodes an aminotransferase critical for synthesizing the biologically active FB_1_ molecule [44]. *FUM1p* has been reported as a major enzyme in fumonisin mediation [80,81]. Considering the effects of ecological factors on gene expression, Lazzaro et al. [82] reported the increased effect of temperature on the expression of *FUM21* than on *FUM2*. *FUM 3* and *FUM 14* have been observed to be affected by water activity in a positive correlation to fumonisin production; *FUM3* increased expression has also been observed to favor the formation of FB_1_ from FB_3_ precursor, possibly due to the role of the gene in hydroxylation of FB_3_ to FB_1_ [83]. Although the exact process involved in the formation of fumonisin is not yet clarified, *FUM1* expression induced as a response to water stress and the rate of fumonisin accumulation affected by water loss during maize ripening suggest fumonisin production results from stress-induced fungal growth [84,85]. The involvement of lipids present in the host plant in the formation of fumonisin has also been reported [86].

The difference in the biosynthesis of fumonisin B1, B2, and B3 is mainly in their hydroxylation patterns, which are primarily determined by the FUM2 and FUM3 genes. When the FUM2 gene is functional, C-10 hydroxylation occurs, leading to the production of FB_1_ and FB_3_. If FUM2 is non-functional, hydroxylation at C-10 does not occur, resulting in the production of FB_2_, which lacks a hydroxyl group at this position [87]. For example, the genome of *A. niger* has eleven *Fusarium* FUM cluster homologues that are made up of the following *Fusarium* genes: fum1 (polyketide synthase), fum3, fum6, and fum15 (hydroxylase), fum7 (dehydrogenase), fum8 (aminotransferase), fum10 (acyl-CoA synthase), fum13 (carbonyl reductase), fum14 (condensation-domain protein), fum19 (ABC transporter), and fum21 (transcription factor) [44]. The *Fusarium* FUM cluster lacks the short-chain length dehydrogenase gene (sdr1), which is present in the *A. niger* FUM cluster, but plays a role in fumonisin production [44]. Additionally, the *A. niger* FUM cluster lacks the *Fusarium* FUM2 gene, which results in fumonisin’s C-10 backbone being hydroxylated [88]. Thus, *A. niger* exclusively produces fumonisins (FB_2_, FB_4_, and FB_6_) when it lacks a hydroxyl at C-10, which is consistent with the absence of a *FUM2* homologue in the *A. niger* cluster [89,90].

## 4. Factors Affecting the Occurrence of Fumonisins

The occurrence of fumonisins in agricultural products is dependent on a range of factors, such as geographical region, season, and particular environmental conditions in which the food product is grown, harvested, and stored. The tropical and subtropical regions of the world, such as sub-Saharan Africa, are the most favorable regions for fungi development on food commodities and mycotoxin production [35,46]. Moisture content and temperature have been demonstrated to be the critical environmental factors that affect the production of fumonisins during storage [91]. The effect of different temperatures and water activities on fungal growth and fumonisin production by *Aspergillus* species was studied by Perera et al. [16]. The study demonstrated the effect of environmental factors on fumonisin production by *Aspergillus niger* and *Aspergillus welwitschiae* at varying temperatures and water activity. The highest growth rate observed was 14.89 mm/day at 0.98a_w_ and 35 °C, with the highest fumonisin production of 25.3 mg/kg observed at 0.98a_w_ and 20 °C for *A. welwitschiae.* In contrast, studies of fumonisin production by *Fusarium* species such as *Fusarium verticillioides* indicate different environmental preferences. For example, an observable increase in FB_1_ production was reported at 15 °C, with a higher fungal growth rate at 25 °C [92]. In another study, Cendoya et al. [93] investigated the effects of abiotic variables, temperature (15, 25, and 30 °C), and water activity (a_W_; 0.995, 0.98, 0.96, 0.94, 0.92, and 0.88) on mycelial development and fumonisin production in three *F. proliferatum* strains isolated from wheat grains in Argentina. They found that the growth rates decreased when the a_w_ of the medium was decreased, reaching their maximum at 25 °C and the highest a_W_ (0.995). Two strains produced the highest amounts of total fumonisins (FB_1_, FB_2_, and FB_3_) at 0.995 a_W_ and 15 °C, while the third strain produced the highest amounts at 25 °C and 0.995 a_W_.

Numerous studies have been undertaken from both natural occurrences and experimental settings on factors affecting fumonisin production. These studies have highlighted the importance of drought conditions in the occurrence of fumonisins. In the planting season of 1993, there were variations in the occurrence of measured fumonisins in corn crops in Ontario, Canada, due to changing rainfall patterns. Areas that received high rainfall (95% of normal value) had low incidences of fumonisin contamination with an average FB_1_ concentration of 0.4 μg/g, while areas with lower rainfall (49% of normal value) had high incidences of fumonisin contamination with an average FB_1_ concentration of 1.4 μg/g [94]. This indicates that drought conditions favor the occurrence of fumonisins in crops. Another study conducted by Kos et al. [95] in Serbia related the increase in *Fusarium* toxins contamination of maize in the farming season of 2012 to drought conditions and the associated increase in temperature. This co-relates with previous data obtained from samples collected in South Africa [96].

Related to drought is the effect of climate change on fumonisin contamination of food and feeds in different parts of the world. Climate is a key agro-system that drives fungal colonization of crops and mycotoxin production [97]. The ability of a fungus to produce mycotoxins is strongly influenced by temperature, relative humidity, and stress conditions of the plant [97,98]. Although different fungal species respond to environmental changes differently, rising global temperature impacts their ability to produce mycotoxins. For instance, *Aspergillus* species are more likely to thrive during post-harvest storage under high humidity, while *Fusarium* species predominantly affect crops in the field, especially during drought conditions. However, both fungal species in these conditions produce mycotoxins. Importantly, this challenge is increasing with the increased rate of warming of the planet in recent years, with the years 2000–2009 reported as the warmest period in recorded history [99]. Furthermore, increased global temperatures also indirectly contribute to fumonisin contamination by exacerbating insect damage to crops, such as kernel damage in corn, which facilitates fungal and increased production/accumulation of mycotoxins [100]. This increase in kernel damage may be because insects are ectotherms and become more active as ambient temperatures rise, leading to an increase in their metabolic and developmental rates and activity patterns [101]. Associated with increased global temperature are other changes in climatic conditions such as variations in rainfall patterns, humidity, drought, atmospheric carbon dioxide, etc. These variations in climatic conditions impact agricultural production and further predispose crops to fungal infection and mycotoxin contamination [102].

## 5. Fumonisins and Climate Change

Climate change factors, including temperature, water availability, and extreme weather events, influence the life cycle of mycotoxigenic fungi and their ability to colonize crops and produce toxins [103]. These changes may lead to shifts in the geographical distribution of *Fusarium* species, expanding the regions at risk of fumonisin contamination. Traditionally, fumonisin contamination has been most severe in tropical and subtropical regions [104]. But recently, rising temperatures have pushed the boundaries of fungal viability into temperate regions, exposing new areas to contamination risks [105]. For instance, parts of Europe and North America that were previously less affected are now reporting increased incidences of fumonisin contamination in maize [106]. Overall, due to the world’s rapidly changing climate and extreme weather events, as well as the associated risks of mycotoxin-mediated food safety and public health issues, a critical understanding of climate-dependent mycotoxin contamination patterns and the ability to anticipate potential climate-induced mycotoxin risks is essential for risk planning, preparedness, and taking a proactive approach in order to minimize human and animal exposure to mycotoxins and limit its socio-economic and health impact [107]. As highlighted, the production of fumonisins is highly influenced by climate factors such as temperature, humidity, and precipitation. Prolonged heat stress can reduce crop resilience, making them more susceptible to fungal colonization and mycotoxins. Altered precipitation patterns also play a significant role. While excessive rainfall can increase humidity levels conducive to fungal growth, prolonged droughts weaken plants, making them more susceptible to fungal infection. An earlier study in the Philippines reported that while increased rainfall may reduce aflatoxin risk in the Philippines, fumonisin risk remains very high under both current and projected climate conditions [108].

Current and projected climate changes are expected to exacerbate conditions favorable for fumonisin production, thereby increasing the risk of contamination in food and feed. Recent studies have provided deeper insights into how current and future climate trends may influence fumonisin contamination in crops. An analysis of sixteen years (2005/2006–2020/2021) of climatic data in South Africa reveals both systematic and erratic variability in critical climatic factors known to influence mycotoxin contamination in crops. Their findings indicate that climatic variability significantly affects fumonisin levels in maize, underscoring the importance of monitoring and modeling climatic patterns to predict and manage mycotoxin risks [107]. Similarly, using a climatic model to predict the occurrence of *Fusarium* toxins in wheat and maize was developed and demonstrated that environmental factors, such as temperature and humidity, accounted for a substantial portion of the variation in toxin levels across different fields [109]. It is known that higher temperatures and prolonged drought conditions significantly increase the susceptibility of crops to fumonisin contamination. During drought conditions, plant stress predisposes crops, particularly maize, to infection by *Fusarium* spp. such as *Fusarium verticillioides*. For instance, a study in Ontario, Canada, observed that areas with lower rainfall (49% of normal) had higher fumonisin contamination levels (average FB_1_ concentration of 1.4 μg/g), compared to areas with higher rainfall (95% of normal), which had lower fumonisin levels (average FB_1_ concentration of 0.4 μg/g) [110]. Similarly, warmer temperatures have been shown to enhance fungal growth rates and fumonisin production, further increasing contamination risks.

Forecasting mycotoxin contamination offers a more effective approach to the control of mycotoxin contamination of foods as it directly addresses food safety and public health risks. Toxin prediction models such as DONcast have been extensively validated and commercialized in Canada [109] and were employed to quantify the variance in toxin levels related to year and agronomic influences on wheat and maize samples collected from agricultural fields. This study found that environmental factors explained 48% of the variation in DON in wheat across all fields, followed by variety (27%), and lastly, preceding crop (14–28%). Up to 80% of the variation in DON was explained by the reliable site-specific DON forecast model, demonstrating the potential of this tool in the qualitative estimation of mycotoxin contamination. The recent study by Gbashi et al. [107] also demonstrated the use of four machine learning models (support vector machines, eXtreme gradient boosting, random forest, and orthogonal partial least squares) in the prediction of mycotoxin contamination patterns in maize in South Africa, which could aid in mycotoxin risk management and control by facilitating early alerts and the adoption of pertinent mitigation measures. Because fumonisins pose serious health risks to humans and animals. Climate-induced increases in fumonisin contamination can exacerbate these public health challenges, particularly in low- and middle-income countries where food safety monitoring systems are weak. Furthermore, food insecurity is likely to worsen as contaminated crops are deemed unfit for consumption, reducing the availability of staple foods with far-reaching economic impacts as contaminated crops fail to meet international trade standards, leading to revenue losses for farmers and exporters. These food safety and security risks associated with climate-induced fumonisin contamination further add to the urgency for sustainability measures to tackle climate change.

## 6. Intake of Fumonisins and Regulations

The daily intake of fumonisins in different food commodities among various countries has been documented [111]. The European Food Standards Agency (EFSA) set up the EFSA panel on contaminants in the food chain (CONTAM), which established health-based guidance values for fumonisins and their modified forms [112]. In the European diet, for instance, the total daily intake of FB1 was estimated to be 1.4 µg/kg of body weight per week by the European Mycotoxin Awareness Network [113]. EMAN proposed a “provisional-maximum-tolerable-daily-intake” (PMTDI) of 2 µg/kg body weight/day for FB1. This was obtained by dividing the “no-observable–effect-level (NOEL mg/kg of body weight/day) by a safety factor of 500. This level was in line with the value set by the World Health Organization’s International Programme on Chemical Safety (IPCS) and the Scientific Committee on Food (SCF) of the European Commission, which, after evaluation of FB1-related risks, set a tolerable daily intake of 2 µg/kg body weight/day for FB1 [114]. In Switzerland, the official tolerance value is 1 mg/kg FB1+FB2 in dry corn products [115]. However, based on the higher prevalence of megalocytic hepatocytes observed in a chronic study involving mice [116], a more recent study by the EFSA Panel on Contaminants in the Food Chain (CONTAM) determined a tolerated daily intake (TDI) of 1.0 μg/kg body weight (bw) per day for FB1 based on a benchmark dose lower confidence limit (BMDL_10_) of 0.1 mg/kg bw per day and an uncertainty factor (UF) of 100 for intra and interspecies variability [112]. In carrying out estimates of mycotoxin intakes in different populations, geographical differences and consumption patterns need to be taken into consideration together with the average weight per person in the group studied [117].

Within the European Union, a maximum limit of 4000 µg/kg for total fumonisins (FB_1_+FB_2_) has been set for unprocessed maize, with an exception for maize intended for wet milling [118]. Maize and maize-based products intended for direct human consumption have a set maximum limit of 1000 µg/kg, while 800 µg/kg is set for maize-based breakfast cereals and snacks. The maximum limit of fumonisins in processed baby food is set very low at 200 µg/kg to protect the health of infants and young children [118,119]. Within the United States of America, the FDA sets out a maximum limit for total fumonisins at 4 µg/g in maize products intended to be used in human foods and 3 µg/g for popcorn grains [120,121]. For animal feeds, the maximum limit ranged from 1 mg/kg in the total diet for horses and rabbits to 50 µg/g for poultry [121].

The largest producer of maize worldwide is the United States of America. In 2012, worldwide production of maize was estimated at 875,226,630 tons, of which the United States produced 31%, China 24%, and Japan produced 8% [122]. However, the regions of the world with the highest production rate are not the highest consumers of the crop. Maize is a staple in many African countries where consumption ranges from 52 to 328 g/person/day, with the highest consumer being Lesotho (328 g), closely followed by Malawi (293 g), Zambia (243 g), Zimbabwe (241 g), and South Africa (222 g). In the Americas, the highest consumption was recorded in Mexico (267 g/person/day) [123]. These regions with the highest consumption of maize have also recorded several cases of disease conditions associated with fumonisins, being linked to neural tube defects along the Texas-Mexico border [5,124].

## 7. Masked/Bound Fumonisins and the Transformation of Fumonisins During Food Processing

Masked mycotoxins are mycotoxin derivatives that are undetectable by use of conventional analytical techniques due to alteration of their structure in the food matrixes they occur in [125,126]. The masking of mycotoxins can be due to chemical transformations catalyzed by plant enzymes involved in detoxification processes [127]. In addition to enzymatic transformations, food processing treatments, including fermentation processes, can also alter the chemistry of mycotoxins. Masked mycotoxins can be in extractable conjugated form or bound non-extractable form, which are covalently or non-covalently attached to polymeric carbohydrate or protein matrices and hence cannot be directly accessible. For their measurement, prior to chemical analysis, they must be liberated by chemical or enzymatic treatment from the sample matrix [128]. Because the masked mycotoxins are not detectable without chemical or enzymatic treatment, analysis of samples that contain these masked mycotoxins usually underestimates the number of mycotoxins present, with bound (non-extractable) mycotoxins completely eluding detection by conventional HPLC methods [127]. Hence the occurrence of covalently bound and non-covalently bound fumonisins in foods is of importance in risk assessment for fumonisins.

There are several reports on the presence of masked fumonisins, which are usually detectable only after alkaline hydrolysis [68,129,130,131]. The mechanism of this masking was initially attributed to the covalent bonds formed between the tricaroxylic moiety and hydroxyl groups of carbohydrates or between the amino groups of amino acids upon application of heat, or amino/sulfidryl groups of the side chains of amino acids in proteins [44,132]. However, it has been demonstrated by Dall’Asta et al. [133] that the masked fumonisins can also appear in maize kernels harvested in commercial fields with modest temperature exposure and not just in thermally treated products. Employing an in vitro digestion model using an enzyme-driven matrix disaggregation methodology to further study fumonisin-matrix interaction in raw maize. Dall’Asta et al. [133] observed a large increase in total detectable fumonisin following digestion of the food matrix, thus indicating the presence of masked fumonisins.

Over the years, bound fumonisins have been reported in various thermally treated food products such as cornflakes [132], gluten-free products [134], heat-processed corn foods [131], noodles, groats, and starch concentrates [135]. The study by Dall’Asta et al. [133] on Italian maize samples measured the presence of free fumonisins in the range of 0.05–40 mg/kg. The samples were then subjected to alkaline hydrolysis to liberate bound fumonisins. Total fumonisin obtained after the alkaline hydrolysis was in the range of 0.05–69 mg/kg, showing a significant increase due to the presence of bound fumonisins.

Several studies have shown that the conversion of masked mycotoxins to their free form during digestion presents a notable risk as they increase the bioavailability of toxic fumonisins beyond what is detectable in the free form [86]. In certain myotoxicity instances recorded, the low mycotoxin content initially found in the corresponding feed did not match clinical findings made in the sick animals. This unexpectedly high toxicity was attributed to the masked conjugated mycotoxin that may have hydrolyzed into the parent toxins in the animal’s digestive tract [136]. This was demonstrated in the study by Gareis et al. [137], where the metabolites zearalenone and α-zearalenol were detected in the feces and urine of the pig fed with a mixed feed artificially contaminated with zearalenone-4-β-D-glucopyranoside. Since the metabolites were not initially detected during routine analysis but were hydrolyzed during digestion, the authors suggested that such masked mycotoxins were possibly involved in the cases of myotoxicity. The European Food Safety Authority (EFSA) highlighted that modified mycotoxins, including masked fumonisins, could hydrolyze back into their parent compounds during digestion and concluded that hidden fumonisins contribute to total dietary fumonisin exposure and pose a significant safety concern, particularly for populations relying on fumonisin-contaminated staples such as maize [138]. Similarly, Bertuzzi et al. found that hidden fumonisins bound to food matrix could be released during digestion, thereby contributing to total fumonisin toxicity. This study underlined the importance of considering hidden fumonisins in risk assessments, as underestimating their contribution could result in incomplete evaluations of fumonisin risks [130]. Based on these findings, the risk level associated with hidden fumonisins is significant enough to warrant concern, particularly in populations with high dietary exposure. However, it is worth noting that the toxicity of masked mycotoxins such as zearalenone and deoxynivalenol varies depending on their chemical properties and exposure levels. The study by Dellafiora et al. [139] investigated the xenoestrogenicity of zearalenone-14-glucoside in comparison to zearalenone and found that zearalenone-14-glucoside can elicit xenoestrogenic responses in vitro, primarily due to its hydrolysis into zearalenone, which binds and activates estrogen receptors, whereas the glycosylated form could not bind and activate the estrogen receptors. Hence, while masked fumonisins might pose potential risks to human or animal health, further research is required to further understand their physiological and toxicological effects, as some of these toxins might not behave like their parent chemicals [136].

## 8. Reduction of Fumonisin Levels in Foods

Food processing treatments such as roasting, frying, cooking, or high-temperature extrusion of corn may result in a reduction of fumonisin concentrations in food products [140,141]. Due to their relative heat stability, fumonisins are only significantly eliminated during operations that involve temperatures above 150 °C [142]. In their study, Jackson et al. [143] investigated the effect of extrusion process on fumonisins using a twin-screw press and observed a 64–72% reduction of fumonisins in extruded corn without glucose and an 89–94% reduction in corn extruded with glucose. In a feeding trial conducted by Voss et al. [144], it was found that nixtamization (alkali cooking) of corn was effective in reducing fumonisin concentrations in contaminated corn with reduced incidences of apoptotic kidney lesions in rats fed the nixtamalized corn version in comparison to those fed corn prepared by conventional means. This shows that nixtamalization is an effective method for the reduction of fumonisins and their toxicity in contaminated corn. Similarly, the study by Xing et al. [145] found that cinnamon oil at a concentration of 280 μg/mL, a temperature of 30 °C, and an incubation time of 120 h significantly led to a 94.06% reduction (from 15.03 to 0.89 μg/mL) in FB_1_ contamination in maize grains. A more recent study by Schambri et al. [146] demonstrated that the initial fumonisin and deoxynivalenol contamination of 1351 µg/kg in maize kernels was reduced by 91% on average after undergoing three popping methods: hot air, hot oil, and microwaves. The hot oil technique appeared to be more efficient, reducing the fumonisin and deoxynivalenol levels by 98% and 58%, respectively.

## 9. Effects of Fumonisins on Humans and Animals

Exposure to fumonisins poses a great risk to human and animal health because of their interference with basic cellular processes, leading to a wide range of adverse effects [1,44]. In humans, exposure to fumonisins is associated with an increased risk of cancers and neural tube defects [147], whereas in animals, it causes severe diseases like leukoencephalomalacia in horses and pulmonary edema in pigs [20]. The impact of fumonisins is varied and usually depends on factors such as the level of exposure, species affected, and specific health conditions, but it generally results in compromised health and increased disease susceptibility across different biological systems [1,147].

## 10. Human Effects

### 10.1. Neural Tube Defects

Neural tube defects are congenital deformities of the brain and spinal cord due to failure in the closure of the neural tube in in-utero conditions [17,148]. There are multiple factors (genetic and non-genetic) that contribute to neural tube defects, one of which is strongly related to maternal folate deficiency in the first trimester of pregnancy. Neural tube defects can have far-reaching consequences such as nerve damage, anencephaly, spinal bifida, partial leg paralysis, stillbirth, or death shortly after birth [147,149]. Fumonisin B_1_ has been implicated in neural tube defects in babies. Fumonisins reduce uptake of folates by disrupting sphingolipid metabolism and consequently folate transport across cell membranes [5,111]. The correlation between fumonisin uptake in diets and incidences of neural tube defects in some populations has been demonstrated in various studies. For instance, Missmer et al. [5] conducted an epidemiological survey on incidences of neural tube defects along the Texas-Mexico border in the United States, correlating this to fumonisin exposure in the mothers using maternal serum measurements of the sphinganine-sphingosine (Sa/So) ratio. The study found a dose-response relationship between maternal fumonisin exposure and increased risk of NTDs in babies, highlighting that fumonisins are potential risk factors for NTD in populations in which maize is a staple diet. One plausible explanation for this phenomenon could be that fumonisin interferes with the high affinity receptor-mediated sphingolipid-dependent lipid raft transport of folate across cell membranes through its inhibition of sphingolipid production [147]. This was demonstrated in an in vivo study by Gelineau-van Waes et al. [150] using a mouse model where increasing doses of FB_1_ were administered to pregnant LM/Bc mice. Results from the study showed that 20 mg/kg FB_1_ at early gestation produced a high incidence of NTDs (79%) in exposed fetuses. The exposure altered the sphingolipid profile and reduced the levels and expression of the folate receptor (Folbp1) in maternal and fetal tissues.

### 10.2. Human Esophageal Cancer and Carcinogenesis

Human esophageal cancer is one of the deadliest cancers worldwide, arising from the oesophagus and the gastroesophageal junction. It can be in the form of squamous cell carcinomas or adenocarcinomas [151]. Various factors have been implicated in the etiology of this cancer, including dietary factors, obesity, and environmental exposure, including fumonisins [152]. The carcinogenic potential of fumonisins is primarily attributed to their interference with sphingolipid metabolism, as well as their ability to induce oxidative stress, apoptosis, and epigenetic alterations, with higher incidence occurring in regions with high maize consumption. FB_1_ inhibits ceramide synthase, a key enzyme involved in sphingolipid biosynthesis. This inhibition disrupts the production of complex sphingolipids, leading to the accumulation of sphinganine and a decrease in ceramide levels. The disruption of sphingolipid metabolism affects key cellular processes, including cellular signaling, membrane integrity, and apoptosis regulation, which can promote carcinogenesis [87]. Furthermore, this disruption of sphingolipid metabolism by FB1 increases production of ROS, which ultimately leads to oxidative stress and lipid peroxidation, with attendant DNA mutation and the development of cancer [153,154]. Similarly, FB_1_-induced disruption of sphingolipid metabolism can trigger apoptosis in certain cell types. The loss of cells through apoptosis may lead to compensatory proliferation of surviving cells, increasing the likelihood of malignant transformation [107]. In addition, recent studies suggest that exposure to fumonisins can disrupt normal DNA methylation patterns, leading to changes in the way genes are expressed. These changes, known as epigenetic modifications, can increase the risk of cancer by turning on genes that promote tumor growth or shutting off genes that protect against it [109].

A study conducted by Sun et al. [155] compared the occurrence of fumonisins in maize samples collected from three different countries in China: Huantai (low incidence of esophageal cancer), Huaian, and Fusui (both with very high incidences of esophageal cancer). The samples were analyzed by use of Enzyme-linked immunosorbent assays (ELISA) and immunoaffinity-HPLC methods. Of the samples from Huantai analyzed, FB_1_ was detectable in 83.3% (40/48) with an average value of 0.65 mg/kg. Samples obtained from Huaian had detectable FB_1_ in 95.7% (112/117) with an average of 2.84 mg/kg, while samples from Fusui recorded 83.0% (78/94) with an average of 1.27 mg/kg. Of the positive samples from the Huaian region, which has a very high incidence of esophageal cancer, 42% (47/112) had FB_1_ levels greater than 2.0 mg/kg in comparison to the 10% (4/40) of the Huantai region with a low incidence of esophageal cancers, suggesting the contributory role of FB_1_ in human esophageal cancers. In a recent study, Yu et al. [156] investigated the mechanisms through which carcinogenic changes induced by FB_1_ occur in human esophageal epithelial cells. It was indicated that with concentrations from 0.3125 to 5 μM, FB_1_ induced cell growth and migration, DNA damage, interference with a protein regulating the cell cycle, and expression of cancerous genes. More importantly, histone deacetylases (HDACs) associated with cancer progression were enhanced, and the PI3K/Akt signaling pathway was activated after treatment with FB_1_.

### 10.3. Acute Mycotoxicosis

Fumonisins have been implicated in incidences of acute human mycotoxicosis characterized by abdominal pain, diarrhea, and borborygmi [157]. An epidemiological survey conducted by Bhat et al. [158] investigating a foodborne disease outbreak in 50 villages in the Deccan plateau of India following consumption of maize, sorghum crops, and unleavened bread found 100% contamination of maize and sorghum samples collected from affected houses with fumonisin B_1_ mycotoxin present within the ranges of 0.14–7.8 mg/kg and 0.25–64.7 mg/kg. In contrast to samples collected from unaffected households, which had lower fumonisin contamination in the ranges of 0.07–0.36 mg/kg and 0.05–0.24 mg/kg. This clearly indicated an association between fumonisin contamination and acute mycotoxicosis.

## 11. Effects in Animals

### 11.1. Equine Leukoencephalomalacia

Equine leukoencephalomalacia (ELEM) is a fatal neurotoxic disease of horses characterized by liquefactive necrotic lesions predominantly in the white matter and, to a limited extent, in the gray matter and cerebrum of the horse brain [159]. Cases of ELEM related to feeding of horses and donkeys with corn contaminated with fumonisins have been confirmed in many regions of the world, such as Hungary, Brazil, and South Africa, amongst others [160]. Onset of the disease can occur 7 days after consumption of the contaminated feed but usually after 14–21 days or rarely 90 days, usually affecting horses in the same farm [161]. ELEM has been experimentally reproduced in donkeys and horses through the feeding of fumonisin-contaminated feeds and purified FB_1_ [162]. It can be deduced that the development of ELEM after oral exposure to fumonisins is dependent on several factors, including length of exposure, level of dietary contamination, susceptibility of animals, and previous exposure [160]. Intravenous administration of fumonisin B_1_ produced a clinical response and disease time course similar to those obtainable in the naturally occurring disease [162]. In a recent study by Reyes-Velázquez et al. [163], the protein concentration in cerebrospinal fluid averaged 197 mg/dL, which is well above the normal range of 20–124 mg/dL obtained in the study by Foreman et al. [162] on FB_1_-induced neurologic disease in horses. Slight elevations in serum protein, the leukocyte count, and creatinine levels point towards disturbed water balance, immune response, potential inflammation, and/or dehydration, which are critical immune and metabolic stress factors that compound into the neurological damage typical of ELEM. However, the diagnosis of ELEM depends on the clinical signs and detection of fumonisin in feed, as it is usually quite difficult to diagnose since the symptoms somewhat mimic other neurological disorders [163].

### 11.2. Porcine Pulmonary Oedema

This toxicosis of pigs results from the consumption of corn feed contaminated by *F. verticillioides* and fumonisins. It is characterized by elevated serum cholesterol and hepatic enzyme levels as well as pancreatic and liver damage. Thousands of pigs died in 1989 from consumption of FB_1_-contaminated feeds [19]. The first symptoms of PPE are feed refusal, followed within 4–7 days by respiratory distress, cyanosis, and death as a result of acute pulmonary oedema and hydrothorax [19]. Porcine pulmonary oedema studies have been conducted through oral and intravenous dosing of swine [19,164]. These studies show that feeding pigs with feeds contaminated with FB_1_ in concentrations ≥92 ppm or ≥16 mg/kg body weight/day led to the development of lethal pulmonary oedema within 4–7 days. A dose-dependent relationship was observed by Zomborszky et al. [165]. Long-term low-dose exposure to fumonisins can lead to non-lethal pulmonary oedema, as reported by Zomborszky-Kovács et al. [166]. Also, hepatic injury characterized by hepatocellular cytomegaly, nodular hyperplasia, and slowly progressive liver disease may occur [167].

The combined administration of *Mycoplasma hyopneumoniae* infection and 20 ppm dietary FB_1_ to female pigs for 42 days in the study by Pósa et al. [168] caused a significant aggravation of pulmonary pathology as judged from gross and pathomorphological examinations. Distinct lung oedema and mild oedema of other organs, besides mild kidney degeneration, were observed in the pigs that were administered FB_1_. Infection with *M. hyopneumoniae* alone resulted in catarrhal bronchointerstitial pneumonia, especially in certain specific lung lobes. Whereas, when the two pathogens are infected, there is exacerbation of pulmonary oedema and irritated bronchointerstitial pneumonia, hence compounding effects of FB_1_ toxicity and respiratory infections in porcine pulmonary oedema.

On the other hand, low-dose dietary exposure to FBs, including doses below EU regulatory limits (maximum of 5 mg FBs (B1 + B2)/kg for complete feed for pigs), was found to induce histological alterations in young pigs [169]. In the study, biochemical and histopathological changes in various organs were recorded in piglets fed with diets containing increasing levels of FBs (3.7, 8.1, and 12.2 mg/kg feed) for 28 days. Results showed that even the lowest tested dose, 3.7 mg/kg feed, significantly increased the sphinganine-to-sphingosine ratio, a biomarker of FBs toxicity, and induced changes in several tissues, including the heart and intestine, whereas higher doses resulted in lesions of the kidney, liver, and lung with symptoms of pulmonary oedema. At the highest dose, exposure to FB resulted in lung lesions characterized by hemorrhage and alveolar oedema-associated porcine pulmonary oedema, thus being in a position to be exacerbated by cardiac strain. These findings point to the fact that even low levels of FB in pig feed can cause widespread organ damage, a fact that underlines the risks of FB contamination below current regulatory limits.

### 11.3. Ruminants

Fumonisin contamination of cattle and other ruminant feeds has been implicated in cases of feed refusal, hepatocellular apoptosis, and severe renal tubular necrosis. These were associated with an increase in Sa and So concentrations in the liver, kidney, lung, heart, and skeletal muscles of the animals [170]. In ruminants, unlike pigs and horses, there was no recorded effect on cardiovascular function [171]. Mathur et al. [170] in a high-dose short-term study in which Holstein milk-fed calves were administered 1 mg FB1/kg body weight intravenously for 7 days observed hepatic and renal damage after 2–4 days of treatment. One-time administration of fumonisins appears to have no detrimental effect on animals, as observed by Prelusky et al. [172], in which cows were administered with 5.0 mg/kg body weight of FB1. The study observed no alteration in plasma Sa or So levels.

Low-dose, longer-term studies conducted by Osweiler et al. [173], which administered a total fumonisin (FB_1_, FB_2_, and FB_3_) concentration of 148 ppm to beef calves for 31 days, observed serum biochemical and histological evidence of hepatic damage. The oral exposure of lambs and goat kids to fumonisin concentrations resulted in renal and mild hepatic toxicity. Edrington et al. [174] recorded death within 7 days in lambs administered with 45.5 mg total fumonisins as a result of renal injury, although Gurung et al. [175] did not observe any clinical signs of toxicity in goats administered with 95 mgFB1/kg of diet for 122 days.

In a recent study, Gallo et al. [176] examined the commonly occurring levels of DON and FB in diets consumed by cows on their performance, dietary digestibility, liver health, and the quality of milk produced. In the study, twelve lactating Holstein cows were fed three diets: a control diet (CTR) with low DON and FB, a mycotoxin (MTX) diet containing moderate levels of mycotoxins, and a mycotoxin diet with the addition of a mycotoxin deactivator (MDP). Results showed that the CTR diet had the highest milk yield at 37.73 kg/day when compared to the MTX and MDP groups. However, the MTX diet diminished the digestibility of the diet, curd firmness, and the expression of immune-related/inflammatory function genes, hence showing an immunosuppressive effect due to FB. Also, an increased level of activities was observed on the liver enzymes following exposure to MTX, a reflection of liver stress. On the other hand, the MDP diet improved nutrient digestibility, indicating that the addition of mycotoxin deactivation products might partly counteract the negative consequences of feeding ruminants with mycotoxin-contaminated feed on health and productivity.

### 11.4. Poultry (Acute Mortality Syndrome)

Fumonisins have been implicated as the cause of the acute mortality syndrome of broiler chickens, which is characterized by a significant increase in mortality in chicks between 10 and 16 days of age [177]. A dose-related increase in mortality of chick embryos and broiler chicks was observed by Javed et al. [178] following dietary administration of pure FB_1_. It should be noted that moniliformin (MON) can also present related symptoms, and in fact, a combinatorial effect of the two mycotoxins has been demonstrated by Ledoux et al. [179]. Studies conducted by Henry et al. [180] utilizing purified FB_1_ incorporated into chick diets (20–80 mg/kg) resulted in increased Sa levels with a corresponding elevation of hepatic enzymes. In similar studies conducted making use of turkey poults, comparable results as those obtained from chicks were observed, although, in the long term, turkeys may be more susceptible to the effects of fumonisins [181]. In studies conducted on ducks, the oral administration of fumonisin-contaminated feeds resulted in hepatic injury at FB1 concentrations of 5 mg/kg body weight after a 12-day feeding period [182]. Tardieu et al. [183] reported a high threshold of ≥10 mg/kg for the elevation of Sa in liver and plasma.

In vitro studies conducted by Qureshi & Hagler [184] and Dombrink-Kurtzman et al. [185] suggest an immunosuppressive effect of fumonisins in poultry, reporting cytotoxicity of FB1 on turkey lymphocytes with a corresponding decrease in the phagocytic potential of chicken peritoneal machrophages. Deshmukh et al. [186] observed that chronic exposure of fumonisins (150 mg/kg) in the diets of quail chicks resulted in increased severity and susceptibility to infections by *Salmonella gallinarium*, with a corresponding increase in diarrhea and mortality amongst the chicks. This may be related to the immunosuppressive effects of the fumonisins.

A recent study in broiler chicks examined the effect of diets with various levels of FB on intestinal gene expression and sphingolipid metabolism, markers of FB toxicity associated with acute mortality syndrome [187]. Broilers were fed from hatch with diets containing 0.4 to 104.8 mg FB/kg until day 20. Dose-dependent alteration of the Sa/So ratio by exposure to FB in all tissues, especially within the kidney, jejunum, and cecum. Of note, slight up-regulation of immune-related cytokine gene expressions was observed in the small intestine, but rather more pronounced at day 10 of the experiment. Furthermore, the cecal tonsils had a biphasic response. Gene expression was not changed dose-dependently; it was, however, more pronounced at 11.3 mg FB/kg. In their 2018 scientific opinion on feed additives containing fumonisins, the EFSA CONTAM Panel established a no observed adverse effect level (NOAEL) for poultry at 20 mg/kg feed [138,188]. Similarly, in their 2022 report, the EFSA CONTAM Panel [189] evaluated the study by Grenier et al. [187] and reported that although Sa/So ratios and expression of immune-related genes were higher at lower levels of 11.3 mg/kg feed, these were not dose-dependent and hence non-adverse or sufficient to establish an NOAEL. However, they considered the decrease in the lipid found in the study by Henry et al. [180] as an adverse effect with which they identified an NOAEL of 20 mg/kg feed. In essence, the studies of Henry et al. [180] and Grenier et al. [187] indicate that while subclinical fumonisin exposure interferes with sphingolipid metabolism and immune signaling, further studies are required to determine their full consequences on poultry health.

### 11.5. Fish

Because maize is a major component of most fish feeds, the toxicity of fumonisins in fish is worthy of mention because of the potential health effects in farmed species. Reports on fish have been controversial. Administration of fumonisin-contaminated feed to adult channel catfish at concentrations ≥10 mg FB1/kg was reported to cause a significant increase in the Sa:So ratio in serum, liver, kidney, and muscles [190]. However, in a feeding study conducted by Brown et al. [191], which fed adult channel catfish with fumonisin-contaminated feeds, the fish studied were able to tolerate up to 313 mgFB1/kg up to a period of five weeks without any histological evidence of toxicity. Whereas in a separate but similar study on adult channel catfish, a concentration of ≥20 mg FB_1_ (from culture material)/kg fed for 10–14 weeks was toxic to the fish, being associated with weight loss, morphological hepatic alterations, and increased susceptibility to the bacterium *Edwardsiella ictaluri*, with death occurring at 320 and 720 mgFB1/kg [192].

A recent study by Lala et al. [193] evaluated the effects of fumonisin exposure on the redox balance of Nile tilapia fingerlings through the measurement of the gene expression of Glutathione peroxidase (Gpx) and heat shock protein (HSP70) in the liver. In the study, a total of 180 fish were exposed to varying levels of FB (0, 20, 40, and 60 mg/kg). Results showed that the GPX expression increased as exposure time increased, whereas HSP70 showed a decrease after 28 days after initially increasing at 14 days, suggesting that GPX and HSP70 were inversely correlated with sub-lethal exposure. These results support the hypothesis that exposure to FB alters the redox-related gene expression, which contributes to oxidative stress responses, immune suppression, impaired growth, and reduction in resilience in fish.

## 12. Methods of Detection

Effective detection methods for fumonisins are very important in light of the health risks posed by these toxins. For food and crop safety, there is therefore a need to develop sensitive, selective, and robust analytical methods that would allow for the reliable detection, accurate monitoring, quality control, and risk assessment of fumonisins. Current detection methods are diverse and include various technologies and approaches that enable the identification and quantification of levels of fumonisin in different samples. While newer colorimetric methods using immunologic and molecular approaches are being developed, including those using dyes, enzymes, aptamers, and even nanomaterials, chromatographic methods coupled with mass spectrometry or other detectors are still the most reliable methods at this present time and, therefore, are the most frequently used analytical method, especially in detailed quantification of fumonisins [194,195].

### 12.1. Immunological Methods

Several immunological methods, such as Enzyme Linked Immuno-Sorbent Assay (ELISA) and flow-through membrane-based immunoassays, have been developed for the rapid determination of fumonisins in foods and feed stuffs [196]. They are based on the recognition of the three-dimensional structure of the fumonisins by a specific monoclonal or polyclonal antibody [197].

ELISAs are simple, inexpensive, and rapid-to-use methodologies, easily adapted for screening purposes, and can be qualitative or quantitative. Direct competitive ELISAs are commonly employed in the analysis of mycotoxins, including fumonisins [198,199]. Various studies have utilized the principle of ELISA for the determination of fumonisins and other mycotoxins in different food matrices [200,201], and a wide range of ELISA kits are commercially available. ELISA methods are usually validated by comparing the data generated from them to LC or GC methods. However, ELISA methods tend to overestimate the concentration of fumonisins in samples [202,203]. This may be due to the cross-reactivity of the antibodies to compounds structurally related to the fumonisins [204]. Furthermore, due to insufficient validation of ELISA methodologies, their use is limited to those matrixes for which they have been validated [198,205]. These challenges highlight the needs for extensive studies into matrix interference, its prevention, and further validation of ELISA methodologies across a range of food and feed matrixes.

Lateral flow immunoassays (LFID) are utilized for the rapid onsite determination of fumonisins. They are based on the migration of the sample along a membrane strip as a result of capillary action and subsequent reaction between immobilized immunoreagents and the mycotoxins [206]. They can be qualitative or used semi-quantitatively and require minimal sample extraction steps. There are widely available commercial products based on LFIDs, and numerous studies have been conducted on the use of such devices in Fumonisin analysis to improve the reliable detection of the mycotoxins in different food and feed matrixes [203,207]. Furthermore, there is a drive for the development of multi-mycotoxin detection dipsticks. Schneider et al. [208] developed a prototype for the detection of Ochratoxin, Aflatoxin B1, Deoxynivalenol, T-2 toxin, Fumonisin B_1_, Diacetoxyscirpenol and Roridin A simultaneously.

A variety of new immunological methods for the rapid detection of fumonisins are emerging. Wang et al. [209] utilized a colloidal gold immunoassay for the determination of FB_1_ contamination with a visual detection limit of 1.0 μg/L FB_1_ using spiked maize samples, which corresponds to a sensitivity of 0.001 ppm in corn. This work was improved on by Molinelli et al. [210], who reduced the assay time from 10 min to 4 min and expanded the methodology to include the quantitative determination of FB_1_, FB_2_, and FB_3_. An ultrasensitive multiplex chemiluminescent biosensor based on enzyme-catalyzed chemiluminescence detection and a highly sensitive CCD camera was described by Zangheri et al. [211]. Their method obtained a detection limit of 6 µg/kg for fumonisins and 15 µg/kg for aflatoxin, which is well below the regulatory limits. Other studies, such as that conducted by Wang et al. [212], utilized fluorescent microspheres (FMs) in labeling monoclonal antibodies for the detection of FB_1_ in maize samples. The method employed obtained a detection limit of 0.12 ng/mL and had a very high recovery rate of 91.4–118.2%. Amongst the immunological methods, ELISA and lateral flow immunoassays remain the most established methodologies for the detection of the fumonisin mycotoxins because of their simplicity, adaptability, and low cost. Commercial immunoassay kits such as Veratox^®^ (Neogen Corp., Lansing, MI, USA) are widely available.

ELISA and LFD primarily target FB1, the most toxic and abundant fumonisin analog. However, these assays often exhibit cross-reactivity with other fumonisin analogs, such as FB_2_ and FB_3_. The degree of cross-reactivity depends on the antibody specificity used in the assay. For example, Wang et al. [209] evaluated a colloidal gold immunoassay for FB_1_ detection and reported moderate cross-reactivity with FB_2_ and FB_3_. The signal intensity for FB_2_ and FB_3_ was lower compared to FB_1_, indicating that these analogs may produce weaker responses in assays designed specifically for FB_1_. Similarly, ELISA-based methods demonstrated varying cross-reactivity with FB_2_ and FB_3_, often requiring calibration curves for each analog to ensure accurate quantification [213,214]. Even though immunological assays are critical for understanding fumonisin distribution on different food matrices, there is a need to validate all fumonisin analogs; otherwise, the resulting data may misinterpret the exposure risk. This is particularly important as FB_2_ and FB_3_, though less toxic than FB_1_, still contribute to cumulative mycotoxin exposure and may have additive effects [215].

### 12.2. Chromatographic Methods and Mass Spectrometry

Chromatographic methods for fumonisin detection are usually coupled to a detector, mainly mass spectrometry (MS) and include thin-layer chromatography (TLC), gas chromatography coupled with mass spectrometry (GC/MS), liquid chromatography-high resolution mass spectrometry (LC-HRMS), and liquid chromatography with mass spectrometric or fluorescence detection (LC/MS) (LC/FLD) [216]. Despite their continuous use, other chromatographic techniques like TLC and GC still have certain limitations when compared to the contemporary LC. Although TLC is a quick, easy, and inexpensive technique that can provide semi-quantitative mycotoxin testing and screening, its resolution is poor, and its identification capabilities are confined to nonspecific approaches [216,217]. On the other hand, GC can offer remarkable resolving power and exceptional peak capacity and can be utilized with specific detectors like electron capture (ECD), and mass spectrometry with both single (MS) and tandem analyzers (MS/MS) [216]. However, mycotoxins are typically nonvolatile and polar substances that require a derivatization step, which may limit their analysis by GC. Hence, LC has become the most beneficial method for mycotoxin analysis as it covers the wide range of molecular structures seen in mycotoxin and provides a variety of stationary phases with distinct chemistry and interaction processes [216]. Furthermore, when used in conjunction with MS, this method (LC-MS) provides unmatched detection and identification capabilities that enable the untargeted investigation of emergent mycotoxins, their metabolites, and novel related compounds, in addition to the detection of well-known mycotoxins with remarkable sensitivity and specificity [216,218].

The reversed-phase elution mode (RP), which uses C18 and C8 stationary phases, is the most often used method in LC analysis of mycotoxins [216]. However, other elution modes like hydrophilic interaction chromatography (HILIC) [219] and/or mixed modes using ion pair chromatography (IPC) and RP can better retain/separate polar and ionizable mycotoxins for accurate detection of fumonisins [216]. Therefore, the elution method, column chemistry, and mobile phase for LC detection of mycotoxins should be carefully selected based on the structure of the analyte.

In the chromatographic methods, the sample preparation steps employed are of paramount importance to achieve correct determination of the concentrations of fumonisins present in a sample. The literature on this topic reveals a range of extraction protocols, including solid-liquid extraction comprising mixtures of organic solvents, usually methanol and acetonitrile, with water and clean-up by solid-phase extraction (SPE) columns before analysis [195,220]. Selective immunoaffinity columns (IAC) are also often employed for sample clean-up. The antibodies immobilized on the IAC beds form a reversible linkage with the mycotoxin molecules, resulting in more efficient elimination of other bound compounds through the washing and elution steps, hence a superior sample clean-up [221]. This methodology was utilized by Solfrizzo et al. [222] in the measurement of fumonisin levels in corn-based infant foods, with recovery rates up to 96% for FB_1_ and 90% for FB_2_. Also, clean-up of samples can be achieved by Anion exchange chromatography [195]. New emerging methods of clean-up involve the use of molecularly imprinted polymeric (MIP) beds, which are filled with synthetic polymers, making them stable and resistant to the influence of various organic solvents, enzymes, and pH [223].

High-resolution mass spectrometry (HRMS), such as orbitrap spectrometers and time-of-flight (TOF) sensors, have recently demonstrated their benefits for retrospective data processing and untargeted compound identification and screening of fumonisins [224]. They have significantly enhanced the detection, quantification, and structural elucidation of fumonisins. HRMS and time-of-flight (TOF) mass spectrometry have emerged as critical tools in mycotoxin research, given that HRMS enables the investigation of complex mixtures, provides mass measurements with a high degree of accuracy and resolution, and allows for the simultaneous determination of several substances. The use of LC-Orbitrap MS is a precise method for detecting both major and minor fumonisin derivatives in food matrices [225]. For instance, Tamura et al. [226] pioneered the use of liquid chromatography-Orbitrap mass spectrometry (LC-Orbitrap MS) to identify fumonisins B-series (FB1, FB2, FB3) and their N-acetyl derivatives (FA1, FA2, FA3) in corn. This study demonstrated HRMSs capability for structural elucidation and detection of modified fumonisins. Righetti et al. [227] tested the versatility of HRMS in detecting and quantifying a broad range of modified mycotoxins. While this study primarily focused on the general applications of HRMS in food contamination research, it highlighted the importance of advanced spectrometric tools, including TOF-MS, in addressing analytical challenges. Most recently, a 2022 study by Zhang et al. [228] developed an ultra-performance liquid chromatography-tandem mass spectrometry (UPLC-MS/MS) method for the quantification of fumonisins B1, B2, and B3 and their hydrolyzed metabolites in broiler chicken feed and excreta. This study demonstrated the method’s high sensitivity and accuracy, underscoring its application in monitoring fumonisins in complex biological samples. Similarly, new insights have been obtained in the study of mycotoxins, their metabolites, metabolism, and bioaccumulation through the coupling of HRMS techniques with metabolomic strategies [224,229]. The study by Wan et al. [230] demonstrated the use of a hybrid ion trap time-of-flight (IT-TOF) for the monitoring of DON and deepoxy-deoxynivalenol (DOM-1) and their derivative in rats and chickens. Similarly, Han et al. [231] demonstrated the use of the combination of liquid chromatography–tandem mass spectrometry (LC–MS/MS) and liquid chromatography coupled with time-of-flight mass spectrometry (LC–TOF-MS) for the determination of the in vivo kinetics and biotransformation of ochratoxin A and its metabolites in rats.

Although fumonisins do not readily absorb UV radiation due to the absence of a UV chromophore, HPLC-FLD is still one of the most employed methods for their detection. Prior to HPLC-FLD analysis, fumonisins are subjected to a derivatization step with *o*-phthalic acid dialdehyde and 2-mercaptoethanol or other reagents [232,233]. Because of the polarity of the fumonisins, their extraction from matrix components is usually achieved by the use of ACN-DW or MeOH-DW mixtures [234]. There have been various comparative studies on the efficiency of using different ratios of these solvents in different sample matrixes with varying results, and this is presented in Table 2, while Table 3 presents different detection methods employed for fumonisins and their limits of detection (LOD).

The difficulty of the accurate analytical determination of fumonisins in samples is compounded by the problem of hidden forms (often referred to as masked mycotoxins) of fumonisins, which are not detectable using the current methods and thus lead to the underestimation of the levels of total fumonisins present [134]. New methodologies are being employed for the measurement of these masked fumonisins. De Girolamo et al. [253] developed an LC-HRMS technique for measuring hydrolyzed fumonisins in maize matrixes by alkali hydrolysis with calcium hydroxide (Ca(OH)_2_) and clean-up with ACN/H_2_O through solid phase extraction (SPE) columns (NH_2_ and C_18_). This methodology was able to detect both FB_1_ and FB_2_ together with their hydrolyzed and masked forms, obtaining mycotoxin levels of 60–5700 µg/kg for fumonisins (sum of FB_1_ and FB_2_), 10–210 µg/kg for partially hydrolyzed fumonisins (sum of PHFB1 and PHFB2), and 30–200 µg/kg for hydrolyzed fumonisins (sum of HFB1 and HFB2). The determination of these forms of fumonisins is important because they are produced mainly during the nixtamalization of maize-based products. Hence, they may go undetected in the routine monitoring of samples for the presence of fumonisins. Dall’Asta et al. [134] employed a multi-residual LC-ESI-MS/MS method for the quantification of free and bound fumonisins in commercial gluten-free food products. Following extraction and clean-up by H_2_O/MeOH and ACN/H_2_O liquid-liquid partition extraction and alkali hydrolysis for free and bound fumonisins, free fumonisins were found to occur in 90% of the samples with an overall median value of 800 µg/kg. It should be noted that even though this level is below the EU legal limit, some of the samples had very high values (up to 3310 µg/kg), which are well above the legal limits. Bound fumonisins occurred in 100% of the contaminated samples with a median value of 148 µg/kg and a maximum value of 1530 µg/kg, with most samples containing higher concentrations of the bound form of fumonisins. Because the bound and free fumonisins have a combinatorial effect, samples analyzed using conventional methods that are considered acceptable based on the set EU limits are found to be contaminated above the limit when concentrations of the bound forms are factored in. Because of the limited diet of people suffering from celiac disease, the incidence of fumonisin concentration in gluten-free food products may be problematic, with an urgent need for more studies in this area.

## 13. Biomonitoring of Fumonisin Exposure

Although acute mycotoxin intoxications do occur with far-reaching impacts, as seen by the 2004 aflatoxicosis outbreak in Kenya [254], chronic exposure to low concentrations of fumonisins is more prevalent and can lead to severe health effects such as increased cancer risk and weakened immunity. Hence, it is important to measure exposure to these mycotoxins. Human biomonitoring (HBM), through the measurement of chemicals or their metabolites in body fluids or tissues, provides a comprehensive assessment of internal exposure to mycotoxins, including fumonisins, by integrating all sources and routes of exposure [255]. Several biomarkers of mycotoxin exposure, including blood and, more commonly, urine biomarkers, exist. However, the development of effective biomarkers for fumonisin, especially the most significant of them (FB_1_), is more complicated [256], especially using urine, which is the most commonly explored bodily fluid for biomarkers and serves as the foundation for fumonisin biomonitoring [257]. According to human validation tests, 0.12% to 2% of FB_1_ is excreted through urine [258,259]. Toxicokinetic studies in rodents reveal that the majority of unmetabolized FB_1_ is retained by the liver and kidneys, with the kidneys more likely to retain more than ten times the amount of fumonisin metabolites [260,261]. Further studies have also shown that FB_1_ taken orally is frequently recoverable from urine and fecal samples, despite being quickly removed from the bloodstream [246,262]. However, it might not be feasible to employ feces in extensive epidemiological research. Similarly, the use of urinary FB_1_ as a biomarker of exposure has the limitation of time, as urinary FB_1_ has a short elimination time of about 5 days [263] and thus cannot be utilized to assay for prior exposures. It poses the further challenge of dietary interference and other allied factors [264,265]. Thus, there is a need for the development and validation of more resilient biomarkers of fumonisin exposure. The initial processes of validating biomarkers involve establishing a good correlation between fumonisin exposure and levels of proposed biomarkers in serum, urine, and/or target organs for objective evaluation [262]. Given the low bioavailability of fumonisins, free FB_1_ and sphingolipid metabolites were evaluated as early biomarkers of fumonisin exposure [266]. Human biomonitoring investigations conducted on individuals in Ghana and Guatemala reported a dose-response connection between urine levels of free FB_1_ and the ingestion of food products contaminated with fumonisin, thereby validating the accuracy of urine-free FB_1_ in tracking food exposures in the human population [257,267].

FB_1_ is characterized by the presence of a sphingoid backbone and can inhibit ceramide synthase through the modulation of two precursors in sphingolipid production: sphinganine (Sa) and sphingosine (So), resulting in an increase in the ratio of Sa/So [268,269,270]. This mechanism, which is the causal pathway of mycotoxins toxicity, is exploited as a biomarker of exposure [256,270]. Thus, sphingoid bases, specifically sphinganine (Sa), sphingosine (So), and their ratio (Sa/So), have become a commonly used biomarker for fumonisin exposure. The Sa/So ratio is a preferred biomarker that may be traced in urine because of the substantial variability of both Sa and So [262]. Their use was validated in the study by Riley et al. [271], in which a relationship between elevated fumonisin excretion and elevated blood levels of sphinganine 1-phosphate and the ratio of sphinganine 1-phosphate to sphingosine 1-phosphate was found. An earlier study by Solfrizzo et al. [272] found that rats given a diet containing 0.5 to 15 mg/kg of fumonisin B_1_ and fumonisin B_2_ for seven days showed a strong correlation between their urine Sa/So ratios and their intake of fumonisins and concluded that the speed and specificity of the Sa/So biomarker method make it an appropriate tool for epidemiological research of fumonisin exposure.

## 14. Conclusions and Future Directions

Fumonisins are ubiquitous contaminants of maize and other types of feeds and foods worldwide. They have been implicated in the etiology of various debilitating animal and human diseases. Hence their detection in both animal feed and food destined for human consumption is of paramount importance to protecting public health. The full impacts of the threat fumonisins pose to humans and animals are not yet fully known; hence, additional effort needs to be made in researching their absorption, metabolism, and factors that affect their bioavailability and toxicokinetics. The methods for detecting this family of mycotoxins continue to evolve. However, fumonisins are often underestimated by analytical methods in heat- or alkali-processed foods because of low recoveries due to the binding of fumonisins to the food matrix or the modification of its structure. Also, fumonisins are often overestimated by immunological methods as a result of cross-reactivity between the antibodies utilized and components of the food or feed matrix under analysis. Efforts should be made into the development of more specific monoclonal antibodies that can be employed in immunoassays and immune-affinity chromatography for more specific binding and quantification of fumonisins in food matrix. This will lead to improved diagnostic capability, especially for farms and onsite processing facilities to segregate and eliminate contaminated food products before they go into general circulation. In addition, research should focus on the determination of substances that interfere with the immunological methods and cause the overestimation of the mycotoxins present and modify the immunoassays accordingly. Finally, because of the growing challenges posed by masked mycotoxins, it is important to explore the development of more implementable analytical methods for the estimation of bound/masked mycotoxins in foods and feedstuffs to reduce their risk in foods destined for human and animal consumption.

## Figures and Tables

**Table 2:** Methods utilized for the extraction of fumonisins.

Food Matrix	Extraction Method	LOD	Reference
Maize	MeOH/H_2_O	2 µg/kg	[235]
Cornflakes	MeCN/MeOH/H_2_O	7.5–20 µg/g	[207]
pig liver	MeOH/H_2_O	0.05 ng/g	[195]
animal feeds and food	MeCN/H_2_O	0.01–0.04 µg/g	[236]
Milk	H_2_O	0.1 µg/kg	[195]
Heat processed corn foods	MeOH/MeCN/H_2_O	0.5 ng/g	[131]
Cereals	MeOH:H_2_O		[237]
Maize	ACN/H_2_O	0.1 µg/kg	[238]
Wheat	ACN/H_2_0/CH_3_COOH	35 µg/kg	[239]
Bread	ACN/H_2_0/CH_3_COOH	8 µg/kg	[220]
Horse feed	ACN/H_2_O/HCOOH	50 µg/kg	[240]
Maize	ACN/H_2_0	100 µg/kg	[241]
Wheat	ACN/H_2_0	20 µg/kg	[242]
Maca	EtOAc/CH_3_COOH	1.0 µg/kg	[243]

**Table 3:** Methods utilized for the detection of fumonisins.

Food Matrix	Detection Method	LOD	Reference
Maize	Solid-phase fluorescence	0.119 µg/L	[244]
Spices and aromatic herbs	HPLC-FLD	40 µg/L	[245]
Body fluids and hair from piglets	LC-MS	0.012 µg/L	[246]
Grains	ICS	5.0 µg/L	[247]
Cereals	Extraction-Immunoassay	5.0 µg/L	[248]
Cereals	Electrochemical immunoassay	0.58 µg/L	[249]
Corn and wheat	Immunoassay	5.23 µg/L	[250]
Corn	Colloidal gold immunoassay	2.5 µg/L	[251]
Traditional Chinese medicinal materials	Multi-IAC and HPLC-MS/MS	0.03 ng/mL	[252]

## Data Availability

No new data were created or analyzed in this study. Data sharing is not applicable to this article.
